# Ethanolic *Citrus sinensis* peel mitigates resorcinol bis(diphenyl phosphate)-induced testicular toxicity in Wistar rats through oxido-inflammatory pathways

**DOI:** 10.5935/1518-0557.20260044

**Published:** 2026

**Authors:** Temilade Olatunji, Adeyemi Fatai Odetayo, Halimat Amin Abdulrahim, Luqman Aribidesi Olayaki

**Affiliations:** 1 Department of Physiology, University of Ilorin, Ilorin, Nigeria; 2 Department of Physiology, Federal University of Health Sciences, Ila-Orangun, Nigeria; 3 Endocrinology, Reproductive, and Metabolism Unit, Federal University of Health Sciences, Ila-Orangun, Nigeria

**Keywords:** flame retardants, orange peel, oxidative stress, inflammation, male reproductive dysfunction

## Abstract

**Objective:**

Resorcinol bis(diphenyl phosphate) (RDP) is a widely used organophosphate flame retardant, yet data on its reproductive toxicity are limited. This study evaluated the gonadotoxic effects of RDP and the protective potential of ethanolic *Citrus sinensis* peel extract (EECS) in male Wistar rats.

**Methods:**

Twenty-eight rats were randomized into four oral treatment groups: control, RDP (500 mg/kg), EECS (600 mg/kg), and RDP+EECS, administered for 14 days.

**Results:**

RDP exposure significantly impaired sperm quality and reduced circulating testosterone levels. Testicular dysfunction was accompanied by qualitative histoarchitectural distortion, oxidative stress, inflammatory responses, and apoptosis. Co-administration of EECS ameliorated these effects, improving sperm parameters and antioxidant status and reducing inflammatory and apoptotic markers.

**Conclusion:**

RDP induces testicular dysfunction through oxidative-inflammatory and mitochondrial pathways, whereas EECS exerts protective effects via antioxidant and anti-inflammatory mechanisms. These findings suggest that EECS may be a promising agent against RDP-induced reproductive toxicity.

## INTRODUCTION

According to the World Health Organization (WHO), approximately 15-17% of couples experience infertility, with male factors contributing to nearly 50% of cases (Eisenberg *et al*., 2023). Despite this, misconceptions persist, with many attributing infertility solely to lifestyle factors or psychological stress (Salvio *et al*., 2022), thereby underestimating factors such as metabolic syndrome, oxidative stress, and genetic predisposition (Mannucci *et al*., 2022). Environmental toxicants also play a significant role, as industrialization increases the release of endocrine-disrupting chemicals that impair testicular function (Fatai & Aribidesi, 2022).

Organophosphate flame retardants (OPFRs) are emerging toxicants of concern due to their ubiquity and potential to disrupt endocrine signaling (Okonofua *et al*., 2022; Zhang *et al*., 2024). OPFRs enhance fire resistance in plastics, electronics, and textiles and can leach into the environment (Ballesteros-Gómez *et al*., 2016). Resorcinol bis(diphenyl phosphate) (RDP), a high-impact OPFR used in electronics, paints, and plastics, has received limited toxicological investigation compared with other OPFRs such as TDCPP and TPP (Ballesteros-Gómez *et al*., 2015; van der Veen & de Boer, 2012; Waaijers *et al*., 2013). Although RDP is considered relatively safe, with low-to-moderate human toxicity (U.S. EPA, 2014), its reproductive effects remain poorly characterized, justifying the current study.

Natural antioxidants, particularly plant-derived flavonoids, have demonstrated protective effects on testicular function (Shirisha *et al*., 2019; Samare-Najaf *et al*., 2023; Pavani *et al*., 2025). *Citrus sinensis* (sweet orange) peel, traditionally considered agro-waste, is rich in hesperidin, naringenin, and polymethoxylated flavones and has been shown to improve spermatogenesis, testicular histomorphometry, and redox balance in multiple preclinical models (Parmar & Kar, 2008; Shirisha *et al*., 2019; Odetayo *et al*., 2025). Therefore, we hypothesized that ethanolic *C. sinensis* peel extract (EECS) would protect against RDP-induced testicular dysfunction via modulation of oxidative stress and inflammatory pathways.

## MATERIALS AND METHODS

### Study Design and Ethical Statement

This study was conducted in accordance with the ARRIVE 2.0 guidelines for reporting animal research. Ethical approval was obtained from the University of Ilorin Ethical Review Committee (Approval Number: UERC/ASN/2024/2982), and the experimental procedures were aligned with the recommendations of the National Institutes of Health for the proper handling of laboratory animals.

### Plant Identification and Extract Preparation

Sweet oranges (*Citrus sinensis*) were collected from Aremu’s farm in Ogbomoso, Oyo State, and identified at the University of Ilorin Herbarium, Department of Biology. A voucher specimen (UIH0001/159) was deposited. The oranges were thoroughly washed and peeled. The retrieved peels were shade-dried (to preserve phytochemical constituents) for 7-10 days and pulverized using an electric grinder. The obtained powder (500 g) was macerated in 2.5 L of 70% ethanol for 72 hours at room temperature with occasional agitation. The extract was filtered using Whatman No. 1 filter paper, and the filtrate was concentrated under reduced pressure using a rotary evaporator at 40-45°C. The concentrated extract was further dried to constant weight (226.5 g) in a desiccator and stored at 4°C in amber bottles. Therefore, the percentage yield was 45.3% [(weight of dry extract / weight of dry plant material) × 100].

### Experimental Animals, Groupings, and Treatment

Twenty-eight male Wistar rats weighing 150-170 g, purchased from Mctemmy Animal Husbandry Farm (BN 2670350), were housed in well-ventilated cages with free access to standard pellet diet and water ad libitum.

The animals were randomly assigned to four groups (n=7 per group) after two weeks of acclimatization, as follows:

- **Group I (Control):** received 5 ml/kg of distilled water.
- **Group II (RDP alone):** animals were exposed to 500 mg/kg of RDP.
- **Group III (EECS alone):** animals were treated with 600 mg/kg of orange peel ethanolic extract.
- **Group IV (RDP + EECS):** animals were co-treated with RDP (500 mg/kg) and orange peel ethanolic extract (600 mg/kg).

All treatments were administered once daily via oral gavage for 14 consecutive days. The sample size was determined assuming a large effect size (Cohen’s d=1.2), a significance level of α=0.05, and 80% statistical power, yielding a minimum requirement of 6 animals per group. This estimate was further supported using the resource equation method, where E=total number of animals - total number of groups (Charan & Kantharia, 2013), ensuring that the error degrees of freedom fell within the acceptable range. To account for potential experimental losses, the calculated sample size (n=6 per group) was adjusted for a 10% anticipated attrition rate, resulting in a final sample size of 7 animals per group (Odetayo *et al*., 2024a; b).

The selected RDP dose (500 mg/kg) is consistent with previous toxicological studies (Liu *et al*., 2023; Heinrich *et al*., 2000), while the orange peel dose is similar to that used by Salah & Abdul-Hamid (2014) and Samuel *et al.* (2019) and is the same as that previously reported by our laboratory (Odetayo *et al*., 2024a; b; Okesina *et al*., 2024a). The 500 mg/kg dose of resorcinol bis(diphenyl phosphate) was selected as a sublethal, biologically relevant dose based on toxicological data indicating rat oral LD50 values above 1,000 mg/kg (Heinrich *et al*., 2000). This dose reliably induces measurable biochemical and histopathological changes without causing mortality while remaining ethically acceptable. The 600 mg/kg dose was selected based on prior toxicological and pharmacological studies demonstrating that citrus peel extracts are well tolerated within the 200-1,000 mg/kg range without observable adverse effects. This dose represents a mid-to-high level that ensures sufficient systemic exposure to bioactive constituents to elicit measurable biochemical and histological responses while remaining within established safety margins.

### Sample Collection and Tissue Processing

Twenty-four hours after the final treatment, the overnight-fasted animals were anesthetized with an intraperitoneal injection of ketamine (40 mg/kg) and xylazine (4 mg/kg), as described by Fatai & Aribidesi (2022). Blood samples were collected via cardiac puncture into plain tubes and centrifuged at 3,000 rpm for 15 minutes to obtain serum for hormonal and biochemical assays. The testes and epididymides were excised. The left testes from each animal were homogenized in phosphate-buffered saline for biochemical analysis, while the right testes were fixed in Bouin’s solution for histopathological examination.

### Hormonal Assays

Serum concentrations of gonadotropin-releasing hormone (GnRH), luteinizing hormone (LH), follicle-stimulating hormone (FSH), and testosterone were determined using specific enzyme-linked immunosorbent assay (ELISA) kits (Elabscience) according to the manufacturer’s protocols. All assays were carried out in duplicate, and the absorbance was read using a microplate reader at specified wavelengths.

### Oxidative Stress, Antioxidant Biomarkers, and Cholinesterase Activity

Testicular reactive oxygen species (ROS) levels were determined as described by Lebel *et al*. (1992). Testicular SOD and GPx (Odetayo *et al*., 2023) were assayed by colorimetric methods as previously reported. Testicular total antioxidant capacity (TAC) was determined as previously reported by Abdelzaher *et al*. (2020).

Plasma acetylcholinesterase (AChE) activity was determined using a sandwich ELISA method. Absorbance was measured at 450 nm using a microplate reader, and concentrations were calculated based on a standard calibration curve.

### Inflammatory Markers

Testicular levels of nuclear factor kappa B (NF-kB), tumour necrosis factor-alpha (TNF-alpha), interleukin-1 beta (IL-1β), interleukin-6 (IL-6), and interleukin-10 (IL-10) were estimated using commercially available ELISA kits (Elabscience, USA). In addition, serum nitric oxide (NO) was estimated as described by Ridnour *et al*. (2000) and Odetayo *et al*. (2023).

### Apoptotic Markers

Testicular levels of cytochrome c were determined using an ELISA method as prescribed by the manufacturer (Elabscience, USA).

### Sperm Profile Evaluation

Epididymal sperm were collected, and sperm count as well as sperm morphology were assessed. Sperm count was determined using a Neubauer haemocytometer. Sperm samples were diluted in phosphate-buffered saline and counted under a light microscope at 400× magnification (Odetayo & Olayaki, 2024).

To assess sperm morphology, a sperm smear was stained using eosin-nigrosin stain. Subsequently, a minimum of 200 spermatozoa per sample were evaluated for morphological abnormalities under oil immersion (1000× magnification). Results were expressed as the percentages of morphologically normal and abnormal sperm.

### Histological Examination

The harvested testes were fixed in Bouin’s solution, dehydrated in ascending grades of ethanol, cleared in xylene, and embedded in paraffin wax. Sections of 5 µm thickness were stained with hematoxylin and eosin (H&E) and examined under a light microscope. Micrographs were captured and evaluated for histopathological changes (Hess & Moore, 1993).

### Statistical Analysis

Data were expressed as mean ± standard error of the mean (SEM). The assumption of equality of variances (homoscedasticity) was evaluated using Levene’s test for homogeneity of variances prior to parametric analysis. The Kolmogorov-Smirnov normality test and one-way ANOVA followed by post hoc Tukey tests were conducted using GraphPad Prism 9 to determine statistical significance between groups (*p*≤0.05).

## RESULTS

### Effect of EECS on Markers of Oxidative Stress

**Figure 1:**
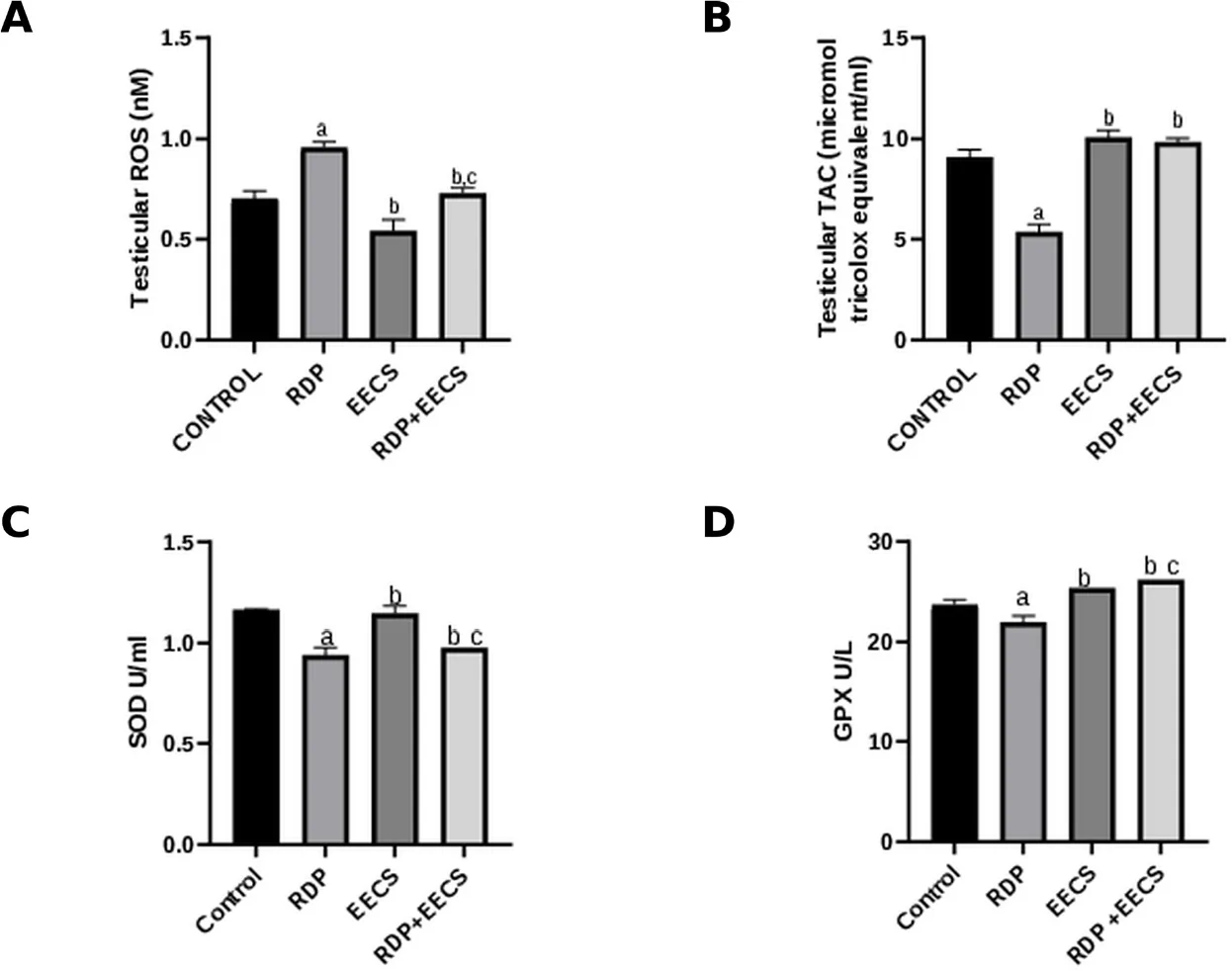
Effect of ethanolic *Citrus sinensis* peel extract (EECS) treatment on testicular oxidative stress markers in RDP-exposed male Wistar rats: (A) reactive oxygen species (ROS), (B) total antioxidant capacity (TAC), (C) superoxide dismutase (SOD), and (D) glutathione peroxidase (GPx). Values are expressed as mean ± SEM (n=7). a *p*<0.05 *vs*. control; b *p*<0.05 *vs*. RDP; c *p*<0.05 *vs*. EECS.

Testicular ROS levels were significantly elevated in RDP-exposed rats (*p<*0.005) compared with controls (Fig. 1A). Co-treatment with EECS markedly attenuated this increase in ROS (*p<*0.001). Similarly, RDP exposure reduced TAC (*p<*0.001) compared with the control group, whereas EECS treatment significantly improved TAC levels (*p<*0.001) (Fig. 1B).

### Effect of EECS on Markers of Inflammation

**Figure 2:**
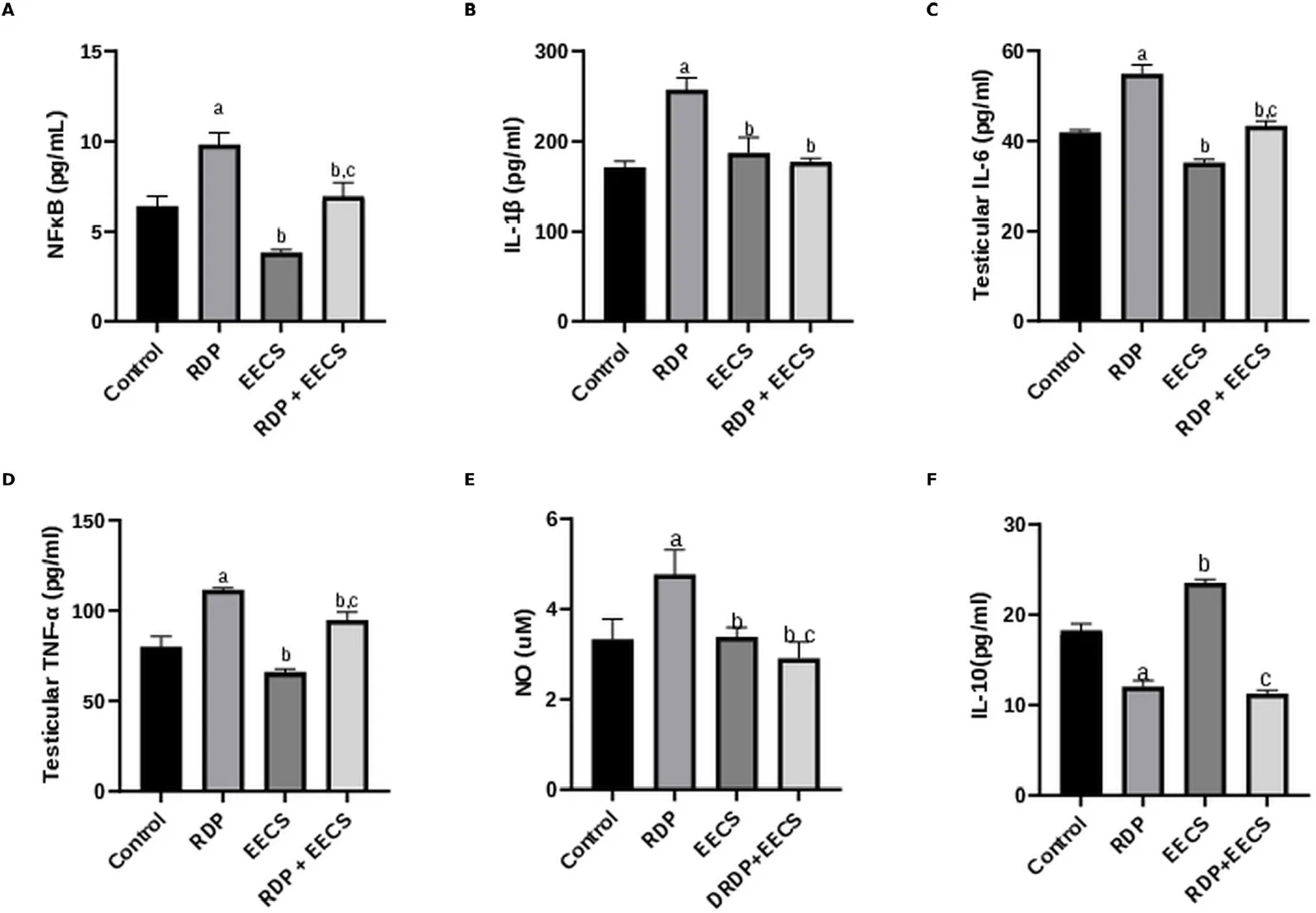
Effect of ethanolic *Citrus sinensis* peel extract (EECS) treatment on testicular inflammatory and nitrosative stress markers in RDP-exposed male Wistar rats: (A) NF-κB, (B) IL-1β, (C) IL-6, (D) TNF-α, (E) nitric oxide (NO), and (F) IL-10. Values are expressed as mean ± SEM (n=7). a *p*<0.05 *vs*. control; b *p*<0.05 *vs*. RDP; c *p*<0.05 *vs*. EECS.

RDP exposure significantly increased testicular NF-kB (*p<*0.007), IL-1β (*p<*0.001), IL-6 (*p<*0.0001), and TNF-alpha (*p<*0.001) compared with controls (Fig. 2A-D). Administration of EECS significantly attenuated these RDP-induced increases (*p<*0.021, *p<*0.002, *p<*0.001, and *p<*0.046, respectively) in testicular inflammatory markers.

### Effect of EECS on Serum Levels of Male Reproductive Hormones

**Figure 3:**
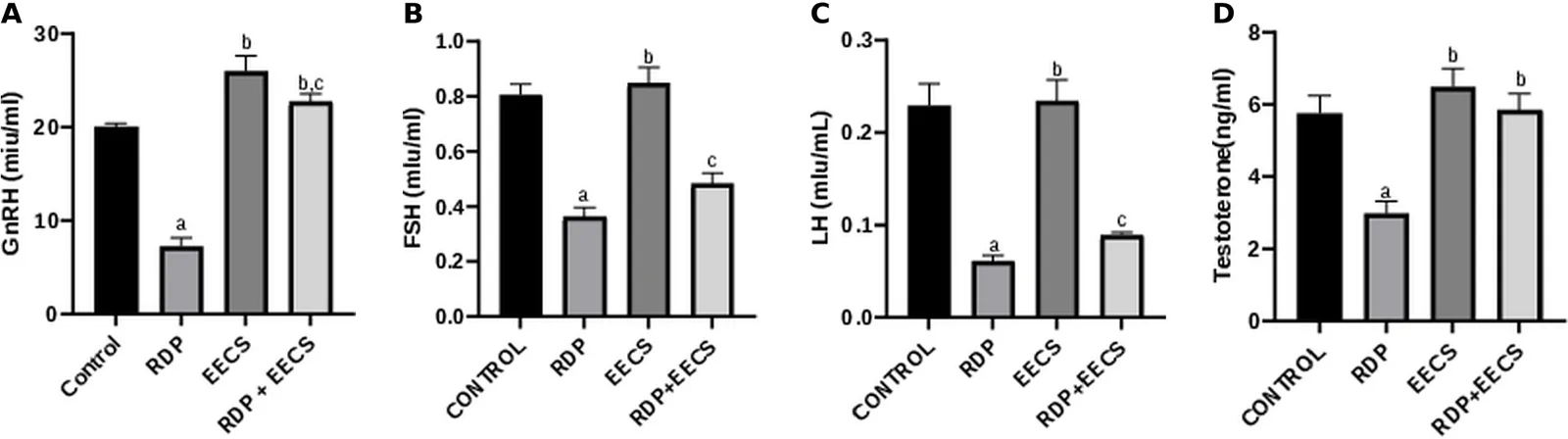
Effect of ethanolic *Citrus sinensis* peel extract (EECS) treatment on reproductive hormone levels in RDP-exposed male Wistar rats: (A) serum GnRH, (B) serum FSH, (C) serum LH, and (D) serum testosterone. Values are expressed as mean ± SEM (n=7). a *p*<0.05 *vs*. control; b *p*<0.05 *vs*. RDP; c *p*<0.05 *vs*. EECS.

RDP exposure significantly reduced serum GnRH (*p<*0.001), FSH (*p<*0.001), LH (*p<*0.001), and testosterone (*p<*0.004) compared with controls. EECS treatment restored GnRH (*p<*0.004) and testosterone (*p<*0.003) levels to near-control values (Fig. 3A, D), but only partially improved FSH (*p<*0.001) and LH (*p<*0.001) levels (Fig. 3B, C).

### Effect of EECS on Acetylcholinesterase (AChE) in Resorcinol bis(diphenyl phosphate)-Exposed Rats

**Figure 4:**
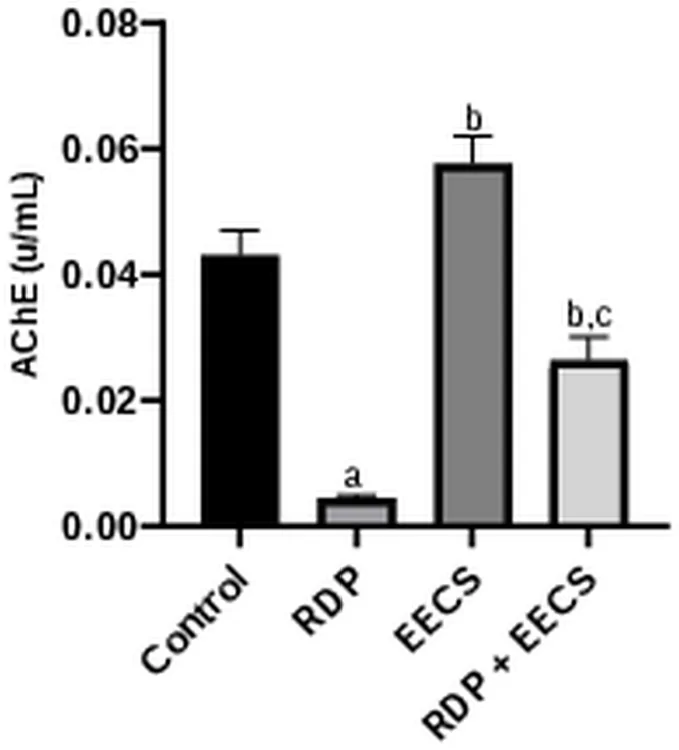
Effect of EECS Treatment on AChE Levels in RDP-exposed Male Wistar Rats. Values are expressed as mean ± SEM (n=7). Where **a** represents *p*<0.05 *vs*. control; **b** represents *p*<0.05 *vs*. RDP; **c** represents *p*<0.05 *vs*. EECS.

The results of this study showed that AChE (*p<*0.001) was significantly reduced in the RDP-exposed group compared with the control group. However, AChE (*p<*0.04) was significantly increased following treatment with EECS (Fig. 4).

### Effect of EECS on Sperm Count in Resorcinol bis(diphenyl phosphate)-Exposed Rats

**Figure 5:**
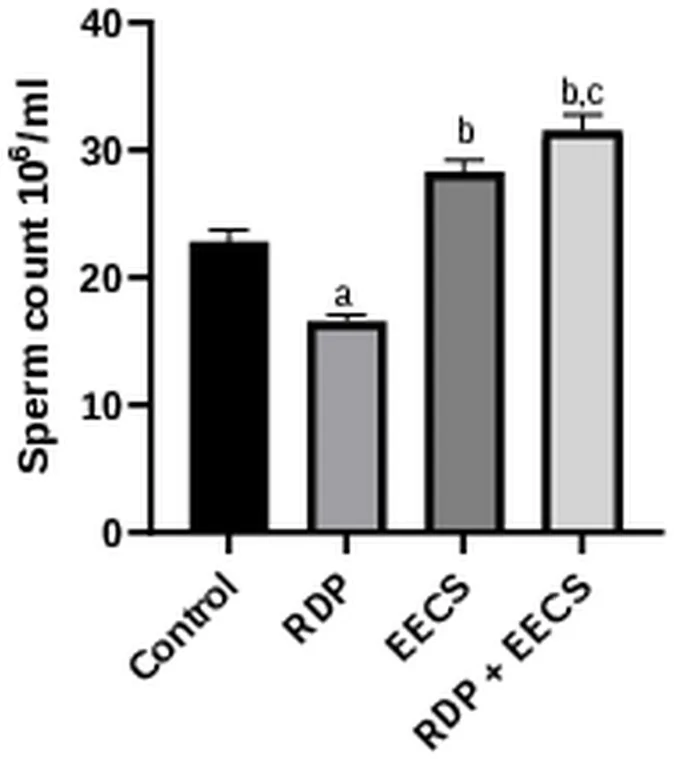
Effect of EECS Treatment on Sperm Count in RDP-exposed Male Wistar Rats. Values are expressed as mean ± SEM (n=7). Where **a** represents *p*<0.05 *vs*. control; **b** represents *p*<0.05 *vs*. RDP; **c** represents *p*<0.05 *vs*. EECS.

Exposure to RDP caused a significant reduction in total sperm count (*p<*0.001) compared with the control group. Sperm count (*p<*0.001) was significantly improved in the group treated with EECS (Fig. 5).

### Effect of EECS on Cytochrome c in Resorcinol bis(diphenyl phosphate)-Exposed Rats

**Figure 6:**
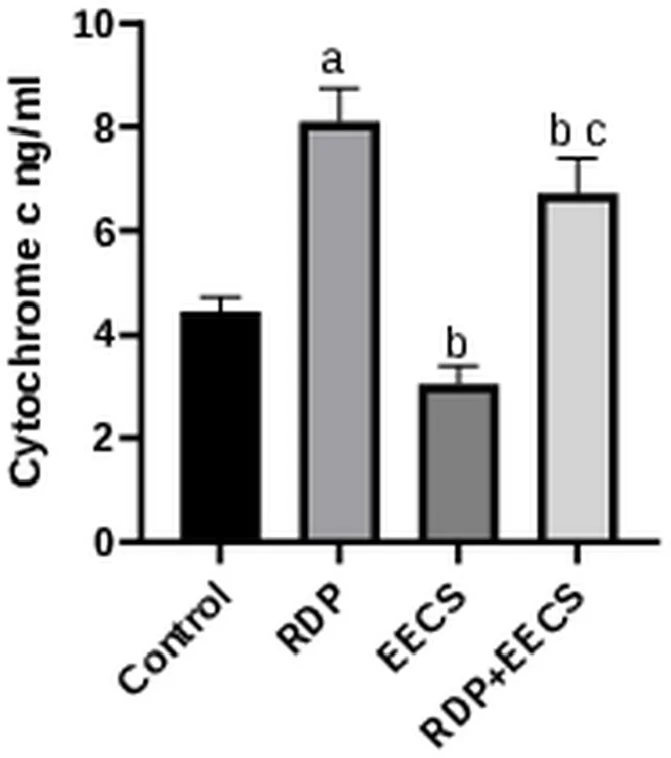
Effect of EECS Treatment on Cytochrome c Levels in RDP-exposed Male Wistar Rats. Values are expressed as mean ± SEM (n=7). Where **a** represents *p*<0.05 *vs*. control; **b** represents *p*<0.05 *vs*. RDP; **c** represents *p*<0.05 *vs*. EECS.

Exposure to RDP caused a significant reduction in cytochrome c levels (*p<*0.001) compared with the control group. Treatment with EECS significantly improved cytochrome c levels (*p<*0.014), indicating a protective effect (Fig. 6).

### Effect of EECS on Histology of the Testis

**Figure 7:**
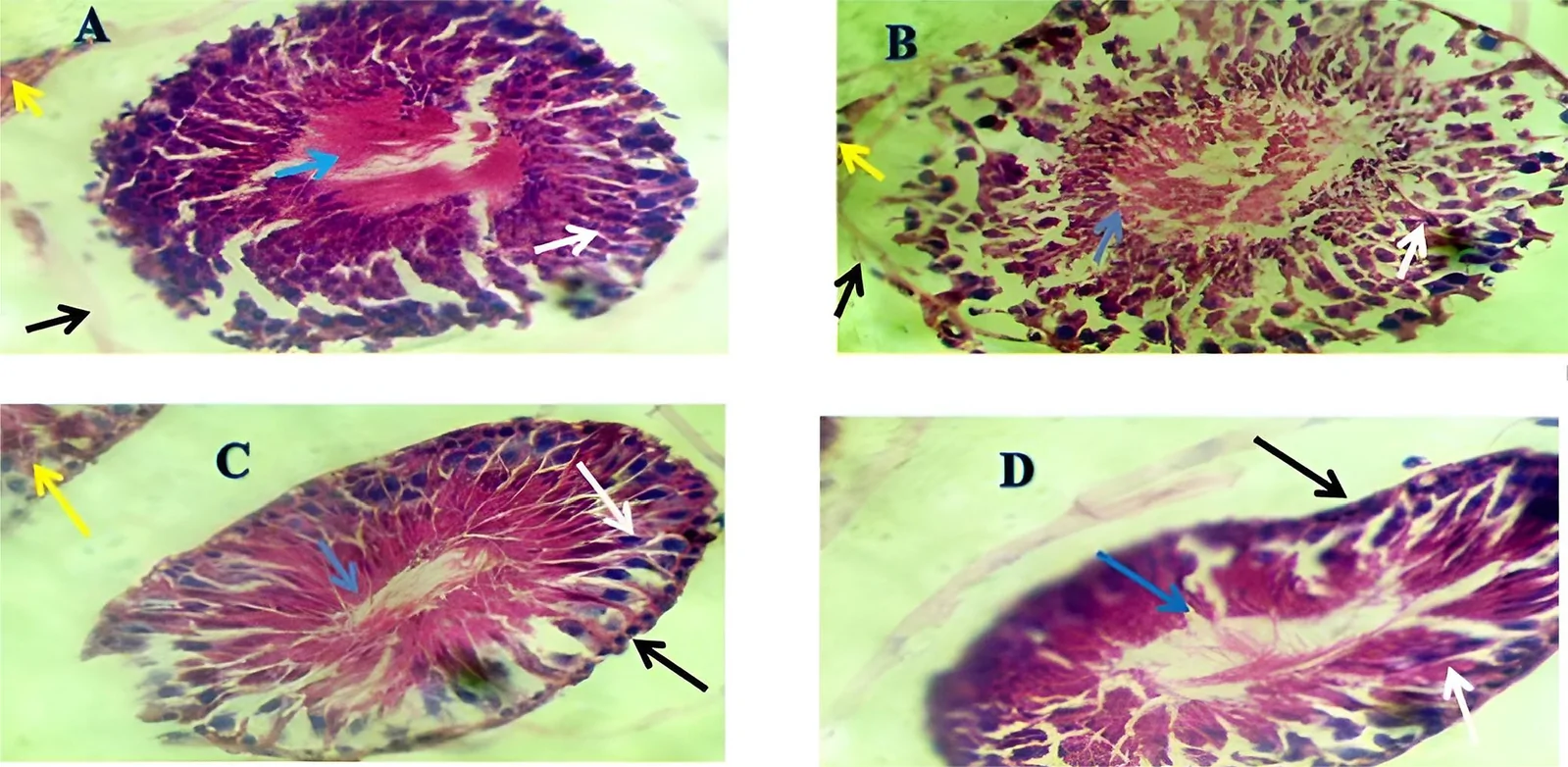
Effect of EECS Treatment on Testicular Histology in RDP-exposed Male Wistar Rats (H&E, Mag.: ×400).

Histological analysis revealed normal testicular architecture in controls, with well-organized germinal epithelium and sperm-filled lumina. RDP administration caused marked degeneration, including disorganized germ cells, sloughing, and reduced luminal sperm (Fig. 7). EECS alone preserved normal histoarchitecture, indicating no toxicity. Co-treatment with EECS partially improved seminiferous tubule organization, enhanced epithelial stratification, and moderately restored luminal sperm, demonstrating its protective effect against RDP-induced testicular damage.

**Plate A (Control):** The seminiferous tubules display normal architecture with well-organized germinal epithelium. The yellow arrow indicates the intact interstitial space with normal Leydig cells. The blue arrow shows mature spermatozoa within the lumen of the seminiferous tubule. The white arrow points to well-aligned spermatogenic cells, and the black arrow highlights an intact basement membrane.

**Plate B (RDP-treated group):** The blue arrow reveals a distorted and reduced spermatozoa population in the lumen. The white arrow indicates disorganization and sloughing of spermatogenic cells into the lumen. The black arrow shows thinning and detachment of the basement membrane. The yellow arrow reflects an edematous interstitial space and possibly a reduction in Leydig cell density, indicating inflammatory changes.

**Plate C (EECS-only group):** Seminiferous tubular architecture is similar to that of the control. The blue arrow shows the presence of mature spermatozoa within the tubular lumen. The white arrow indicates a well-organized germinal epithelium. The black arrow confirms an intact basement membrane, and the yellow arrow shows healthy interstitial tissue, suggesting that EECS alone has no adverse effects and may support testicular integrity.

**Plate D (RDP + EECS group):** The blue arrow denotes the reappearance of spermatozoa in the lumen, albeit in reduced quantity. The white arrow points to partially improved spermatogenic layers, while the black arrow shows improved basement membrane integrity. This suggests a partial ameliorative effect of EECS against RDP-induced testicular damage.

## DISCUSSION

The available literature on the toxic effects of RDP is relatively sparse compared with that on other OPFRs. Given the increasing quantity of RDP and its breakdown products in the environment, as well as growing evidence linking environmental toxicant exposure to male infertility, it is necessary to examine the effects of RDP on testicular toxicity (Ballesteros-Gómez *et al*., 2015; Zhang *et al*., 2024). In the present study, RDP administration significantly disrupted the redox milieu of the testes, as evidenced by reduced TAC, increased ROS concentration, and decreased enzymatic and non-enzymatic antioxidant activities. In addition, testicular inflammatory markers such as NF-kB, TNF-alpha, IL-6, and IL-1β were significantly upregulated, leading to substantial disruptions in the normal concentrations of GnRH, LH, FSH, and testosterone, as well as sperm quality. Testicular toxicity was also confirmed by decreased sperm count and distortion of testicular histology. Conversely, administration of EECS ameliorated testicular toxicity by effectively improving circulating hormone levels and suppressing oxidative stress and inflammatory responses.

In the testis, the balance between ROS and antioxidant defence is essential for proper steroidogenesis and germ cell support (Asadi *et al*., 2017). The mitochondria, which are responsible for supplying the energy used by testicular cells through the electron transport chain, generate small amounts of ROS as by-products. Under physiological conditions, this ROS is scavenged by antioxidants present in the cell (Schang *et al*., 2016). However, exposure to toxicants disrupts this normal process, leading to an overproduction of ROS that overwhelms the antioxidants present in testicular tissue (Darwish *et al*., 2025). RDP administration in this study followed a similar trend, as evidenced by increased ROS and depleted TAC. Although previous studies have not examined the direct effects of RDP on oxidative stress, OPFRs, as reported by Schang *et al.* (2016), disrupt mitochondrial membrane potential. This disruption can, in turn, impair ATP synthesis, propagate oxidative stress, and initiate apoptotic pathways. Further research by Chen *et al*. (2015) demonstrated that mice exposed to TPP and TCEP showed reduced glutathione concentration, as well as reduced glutathione peroxidase (GPx) and catalase activity, thereby permitting the accumulation of ROS. Sustained oxidative stress (increased ROS and decreased TAC) in the testicular microenvironment is particularly deleterious, as spermatogenic cells are highly susceptible to peroxidative damage, which disrupts sperm membranes, proteins, and DNA and ultimately results in reduced sperm concentration (Chen *et al*., 2015; Chen *et al*., 2020). Additionally, AChE activity was significantly reduced following RDP exposure. This RDP-induced reduction in AChE could be linked to its structural similarity to classical organophosphate pesticides, which are known inhibitors of AChE (Chen *et al*., 2015).

Therefore, inhibition of AChE results in unchecked cholinergic signalling due to overstimulation of cholinergic receptors, leading to abnormal calcium influx and activation of signal transduction pathways that converge on mitochondrial dysfunction (Hamed *et al*., 2023; Schang *et al*., 2016) and oxidative stress (Odetayo & Olayaki, 2023). Therefore, the observed decrease in AChE contributes, at least in part, to the redox imbalance observed following RDP exposure. In addition to inducing testicular damage, RDP can also impair testicular function by disrupting hormonal balance. OPFRs are recognized endocrine disruptors, and while RDP has not yet been directly linked to this effect in previous studies, its breakdown components and impurities, such as TPHP, para-OH-TPHP, and resorcinol, are known endocrine disruptors (Ballesteros-Gómez *et al*., 2015; Dishaw *et al*., 2014). Consistent with previous findings, our study showed significant disruption in the levels of circulating male sex hormones (such as GnRH, LH, and FSH), which are responsible for maintaining testosterone production. Therefore, the RDP-induced decrease in testosterone synthesis could result from either its direct effect on the testis or its endocrine-disrupting activity.

The dysregulation of the endocrine output of the testis also has downstream effects on spermatogenic processes, eventually leading to reduced sperm quality and quantity. In the current study, we showed that sperm count was drastically reduced after RDP exposure. Studies by Chen *et al*. (2015) and Pyambri *et al*. (2025) also support this finding. Lahimer *et al*. (2023) noted that testosterone is essential for the proper development of spermatozoa, and reduced sperm quantity can be associated with reduced testosterone secretion as well as disruption of the hypothalamic-pituitary-gonadal axis, as observed in this study.

However, our findings showed that EECS was able to reduce ROS levels and increase TAC. The ability of EECS to restore TAC and reduce ROS is central to its therapeutic efficacy (Abou Baker *et al*., 2022; Liew *et al*., 2018; Shirisha *et al*., 2019). Citrus peels have been shown to be particularly rich in flavanones such as hesperidin, rutin, quercetin, and naringenin, which possess potent free-radical-scavenging abilities due to their phenolic hydroxyl groups (Akunna *et al*., 2017; Olayaki *et al*., 2023). When administered alone, EECS did not cause any deleterious changes and maintained parameters similar to those of the control group. Its bioactive phytochemical constituents counteracted RDP-induced oxidative insults by neutralizing ROS and restoring the balance of antioxidant defences (Abou Baker *et al*., 2022). Odetayo *et al*. (2024a; b) noted that these flavonoids help elevate TAC in testicular tissue by directly donating electrons to free radicals, thereby protecting lipid membranes and mitochondrial integrity from peroxidative damage. Moreover, EECS significantly attenuated the inflammatory response, as demonstrated by the significantly reduced concentrations of NF-kB, TNF-alpha, IL-6, and IL-1β. Since EECS targets ROS buildup in the tissue and mitigates it, there is concomitant inhibition of the redox-sensitive transcription factor NF-kB (Okesina *et al*., 2024a). As noted earlier, activation of NF-kB propagates secretion of other inflammatory cytokines, including TNF-alpha, IL-6, and IL-1β, upon translocation to the nucleus (Darwish *et al*., 2025), all of which were attenuated by treatment with EECS. Studies have also noted that the antioxidant constituents of EECS do not merely scavenge free radicals but also directly interfere with signalling molecules that contribute to the inflammatory pathway (Ademosun *et al*., 2019). These antioxidative and anti-inflammatory properties of EECS are particularly important because the released cytokines do more than induce local inflammation; they also trigger apoptotic pathways that can result in germ cell death and impaired spermatogenesis (Akunna *et al*., 2017; Odetayo *et al*., 2024a; b).

Consequently, preservation of the testicular microenvironment through EECS’s antioxidant and anti-inflammatory actions directly impacts sperm parameters and steroidogenesis, as shown in this study by improved sperm count and testosterone production. By alleviating the increased levels of ROS, testicular cells, including Leydig and Sertoli cells, are spared, thereby preserving their normal function (Salah & Abdul-Hamid, 2014). For Leydig cells, maintenance of cell membrane integrity and mitochondrial function ensures that testosterone production proceeds normally. In addition, ROS scavenging by EECS prevents oxidative modification of key protein enzymes necessary for testosterone production (Liew *et al*., 2018; Shirisha *et al*., 2019). Furthermore, because testosterone production is normalized, the HPG axis receives accurate feedback regulation from the testis, thereby correcting abnormal hormone secretion (Odetayo *et al*., 2024a; b). The result is normalization of sperm parameters, such as sperm count.

In agreement with our study, other experimental models of toxicant-induced testicular damage (e.g., cadmium, tramadol, or streptozotocin) demonstrated that administration of EECS resulted in significant improvements in multiple parameters, including antioxidants, inflammatory cytokines, normalization of AChE activity and the HPG axis, and sperm quality and quantity (Akunna *et al*., 2017; Shirisha *et al*., 2019; Okesina *et al*., 2024b). In addition, in the group treated with EECS, the extract mitigated degenerative changes within the testicular architecture, as evidenced by improved seminiferous tubule morphology, increased thickness of the germinal epithelium, and improved integrity of Leydig cell populations. These histological changes are likely mediated by the combination of reduced oxidative stress, anti-inflammatory signalling, and normalization of endocrine function (Ademosun *et al*., 2019; Abou Baker *et al*., 2022; Odetayo *et al*., 2024a; b).

## CONCLUSION AND FUTURE PERSPECTIVES

This study demonstrates that RDP induces testicular dysfunction through oxidative stress and inflammatory pathways, while EECS confers protective effects. However, further mechanistic investigations and dose-response studies are necessary to establish translational relevance. Additionally, testicular histology was assessed qualitatively without quantitative evaluation using indices such as Johnsen’s score. Future studies should incorporate standardized histomorphometric analyses to strengthen the robustness and interpretability of the findings.

## List of Abbreviations

EECS: ethanolic extract of *Citrus sinensis* peel

FSH: follicle-stimulating hormone

GnRH: gonadotropin-releasing hormone

GPx: glutathione peroxidase

LH: luteinizing hormone

OPFR: organophosphate flame retardants

PBDEs: polybrominated diphenyl ethers

RDP: resorcinol bis(diphenyl phosphate)

ROS: reactive oxygen species

SOD: superoxide dismutase

TAC: total antioxidant capacity
